# Impact of Notches on Additively Manufactured Inconel 718 Tensile Performance

**DOI:** 10.3390/ma16206740

**Published:** 2023-10-18

**Authors:** Joseph Johnson, Daniel Kujawski

**Affiliations:** Mechanical and Aerospace Engineering, Western Michigan University, Kalamazoo, MI 49008, USA; daniel.kujawski@wmich.edu

**Keywords:** laser powder bed fusion, Inconel 718, tensile, notches, notch sensitivity

## Abstract

This study was completed in effort to characterize the notch sensitivity of additively manufactured (AM) Inconel 718 produced by laser powder bed fusion (L-PBF). Three different root radii on V-notched test specimens and smooth specimens were evaluated under tensile conditions for specimens built in vertical and horizontal orientations. Both the total axial strain and localized notch diametral strain were measured. Finite element analysis (FEA) was completed on each specimen geometry to confirm the actual strain measurements near the notch. Test results showed the tensile strength of the notched specimens were larger than the tensile strength values of the smooth specimens. These tensile results equate to a notch-sensitivity ratio (*NSR*) greater than one, indicating that the L-PBF Inconel 718 material is a notch-strengthened material. It is suspected that the notch strengthening is a result of increased triaxial stress produced near the notch tip causing added material constraints, resulting in higher strength values for the notched specimens. Fractography analysis was completed on the various fracture surfaces and identified a dominate ductile failure mode within all of the specimens; however, the amount of ductility reduced with smaller notch root radii. While this study provides the initial notch responses of the L-PBF Inconel 718, further research must be completed in regard to the impact of notches on more complex loading behaviors, such as fatigue and stress-rupture conditions.

## 1. Introduction

Additive manufacturing (AM) has been used to manufacture near-fully dense metallic-production hardware by using a layer-by-layer build approach. There are various methods of producing metal components from AM; however, one of the most common is laser powder bed fusion (L-PBF) [1,2]. L-PBF has grown in popularity for use in production settings due to its ability to consistently produce production hardware with material and mechanical properties similar to conventionally produced parts. The L-PBF process provides an opportunity to design and produce complex geometries with minimum waste, which could not be constructed with conventional manufacturing methods [2]. These now-possible complex geometries allow for increased design freedom to provide increased component performance and cost-saving opportunities [2,3,4]. These benefits are of great interest for fields such as the aerospace and gas turbine industry due to the ability to increase performance, quality, and reduce cost [5]. However, it is noted that L-PBF technology does come with limitations and must be fully understood prior to implementation. Due to the limited amount of available material datasets on metallic materials produced by L-PBF processes, there remains a great deal of uncertainty around the technology for use in critical components. A complete understanding of the applicable material and mechanical properties produced from L-PBF processing must be fully understood prior to implementation into critical components [2,3,6].

One such area of interest that may be directly impacted from the L-PBF build process and its parameters is the notch sensitivity of the L-PBF-produced material [6,7,8,9]. The understanding of notch sensitivity is extremely important when considering designs that may have notches, threads, or inherent flaws. These locations are considered to be stress risers and must be considered during the assessment of a product design. Since L-PBF has the potential for producing inherent flaws, understanding the notch sensitivity of L-PBF-produced material, and how it may differ from more conventionally produced materials, is critical to appropriately predict the life of the product [2,10,11]. Furthermore, L-PBF materials add an additional degree of complexity due to their potential for anisotropic material behavior with respect to the build orientation [12]. A variety of studies concluded differing results of notch sensitivity when comparing different L-PBF materials with their conventional wrought form [13,14]. As described in prior work, there is not a general rule for determining the influence of notches on the material performance for any given material; however, this paper outlines the methods used in prior works [15].

This study focuses on the effect of notches on Inconel 718 produced by L-PBF. The results presented within this study can be used to ensure that notched-type design features, such as threads or shoulders, can withstand the loading conditions that the product sees in service. Included in the evaluation is the influence of build orientation to determine if there is an anisotropic response to the effect of notches. Three different root radii on V-notched test specimens and smooth specimens were evaluated under tensile conditions at room temperature. Resulting tensile performance and fracture surfaces were evaluated and compared to the baseline smooth specimen. Finite element analysis (FEA) was completed on each specimen geometry to validate the actual strain measurements near the notch. In addition, a Ramberg–Osgood relationship was developed for the smooth specimen data and used to calculate the localized stress within the notch via Neuber and Glinka approximations. The calculated approximations were then compared to the actual test data.

## 2. Materials and Methods

**Figure 1:**
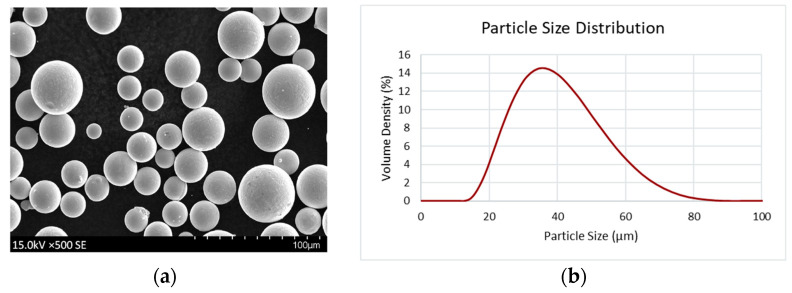
(**a**) Scanning electron microscope (SEM) image of representative L-PBF Inconel 718 powder; (**b**) L-PBF Inconel 718 powder particle size distribution.

For this study, Inconel 718 specimens were manufactured by L-PBF on a Concept Laser Series 5 machine. The powder used during this study was produced from wire feedstock using a plasma arc atomization process within an argon atmosphere. The chemical composition of the L-PBF Inconel 718 powder certified by the supplier is presented in Table 1. Figure 1a provides a representative SEM image of the L-PBF Inconel 718 powder, and the particle size distribution measured per ASTM B822 is shown in Figure 1b [16]. It is noted that the morphology of the powder used is uniform and spherical with little-to-no irregularities. The powder size distribution shows that most of the particles used are under 80μm in diameter, with the highest volume density around 35 μm. In addition to the chemical composition and particle size distribution, the powder skeletal density was measured per ASTM B923 and the powder tap density was measured per ASTM B527 [17,18]. The resulting skeletal and tap density measurement for the powder are 8.44 g/cc and 5.25 g/cc, respectfully.

**Figure 2:**
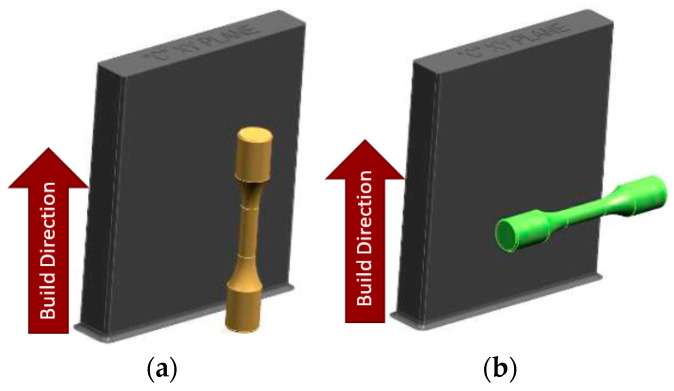
Material specimen blocks showing: (**a**) Vertical specimen; (**b**) Horizontal specimen.

A variety of raw material blanks were printed using a nitrogen cover gas on the Concept Laser Series 5 machine. Figure 2 shows the material blocks, which includes (a) vertical material blocks and specimens, and (b) horizontal material blocks and specimens. Each block provided enough material to machine either 6 vertical specimens or 6 horizontal specimens. 

After the L-PBF process, the material was stress-relieved on the build plate at 954 °C for 2 h and then sectioned off the build plate using a wire EDM. After the parts were removed from the build plate, the material blanks went through a hot isostatic pressing (HIP) operation at 1162 °C and 100 MPa for 4 h. Following the HIP process, the specimens were solution-annealed and precipitation heat-treated. The times and temperatures for both the solution-anneal and precipitation heat-treat process were determined from [19,20,21]. The material was solution-annealed at 980 °C for 1 h and then followed by a two-stage precipitation heat-treatment. The 1st stage of the precipitation heat-treatment was conducted at 720 °C for 8 h, followed by the 2nd stage, which was conducted at 620 °C for 8 h.

**Figure 3:**
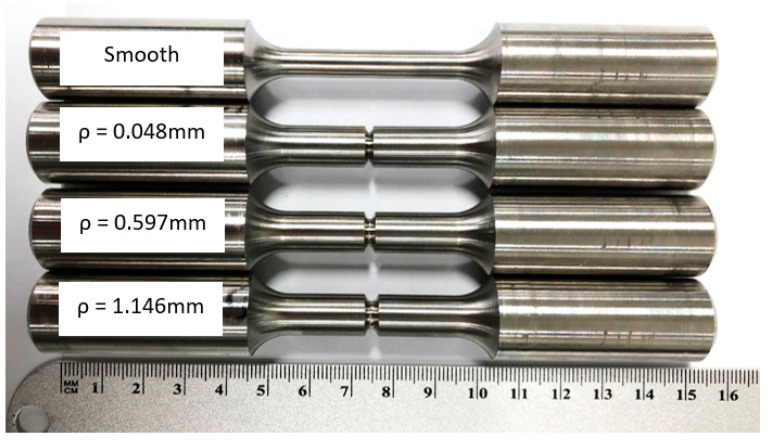
L-PBF Inconel 718 tensile smooth and notched specimens.

**Figure 4:**
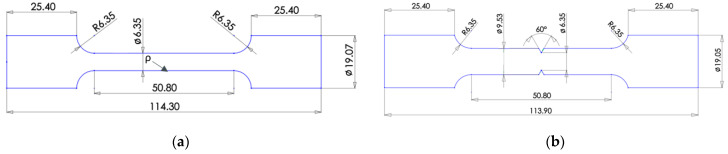
Specimen geometry with dimensions in mm: (**a**) Smooth specimen geometry; (**b**) Notched specimen geometry.

The specimens shown in Figure 3 were machined out of the blocks, as shown in Figure 2. Four configurations of the tensile specimens were machined. Standard smooth tensile specimens were produced with reference to ASTM E8 [22]. Notched tensile specimens included three different notch root radii, *ρ*. The notch root radii included values of 0.048 mm, 0.597 mm, and 1.146 mm. The gauge diameter for the smooth specimen was designed at 6.35 mm, as shown in Figure 4a, while the notch specimens were designed with a minor notch diameter of 6.35 mm and a major notch diameter of 9.53 mm, as shown in Figure 4b.

**Figure 5:**
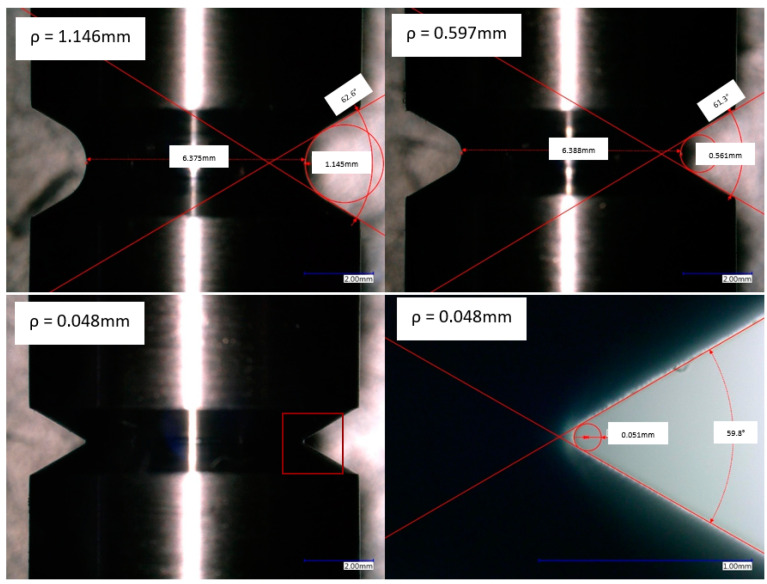
Angle and radii measurements of the notched specimens.

The notch geometry of each specimen was measured and verified using a Keyence VHX 6000 digital microscope. Representative images of each notch geometry are shown in Figure 5.

Five tensile specimens were tested for each specimen geometry and build orientation. All specimens were tested under displacement-controlled conditions at room temperature with a displacement rate of 0.140 mm/s until failure. Three of the specimens were tested using Western Michigan University’s MTS 810 hydraulic machine with an axial extensimeter. The remaining two specimens were tested at Westmoreland Mechanical Testing & Research, Inc. (Youngstown, PA, USA) utilizing a diametral strain gauge. Table 2 provides the entire tensile test matrix, including specimen type, specimen build orientation, strain measurement method, and test quantity.

## 3. Results

### 3.1. Stress vs. Strain Curves

**Figure 6:**
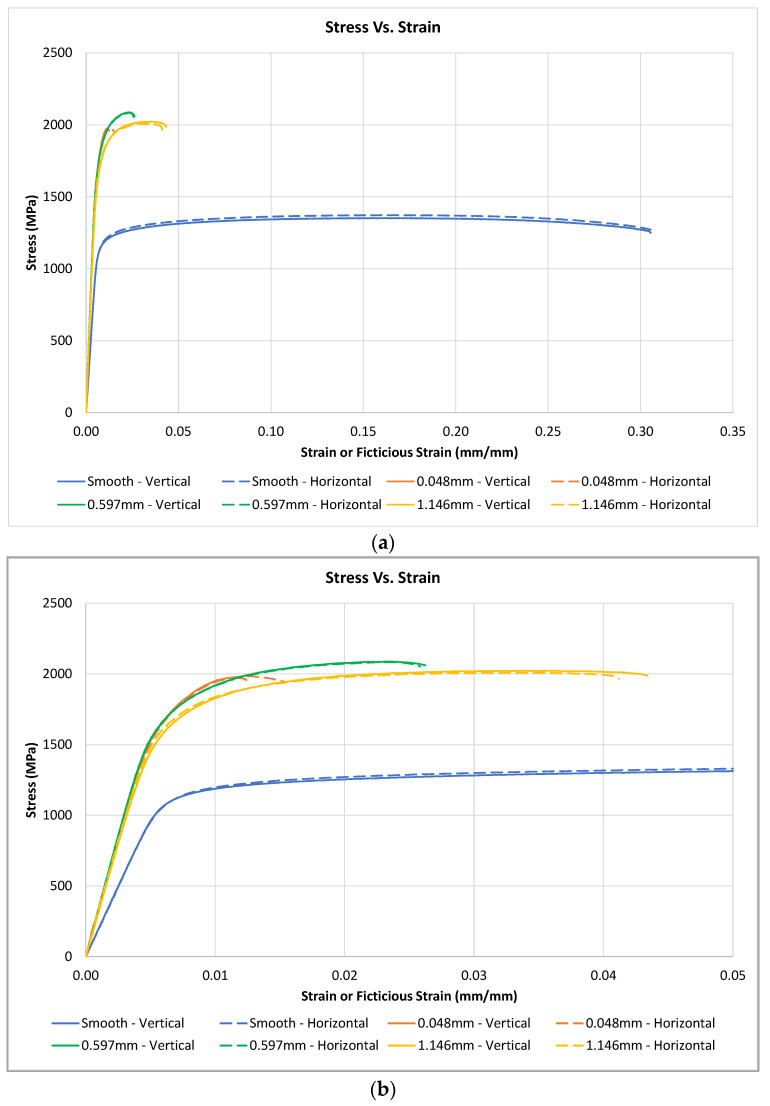
Stress vs. axial-strain curves for smooth and notched specimens machined in the vertical and horizontal build directions: (**a**) Entire-strain or fictitious-strain scale; (**b**) Reduced-strain or fictitious-strain scale to evaluate differences within the notched specimens.

A typical stress vs. strain curve for each specimen type, tested with axial-strain measurements, is plotted on the stress vs. strain curves shown in Figure 6a,b. It is noted that the stress values for the smooth specimens were calculated using the 6.35 mm gauge diameter cross-sectional area, while the stress values for the notched specimens were calculated using the 6.35 mm notch minor diameter. Differences between the notched and smooth specimens’ tensile performance can be noted in the calculated modulus of elasticity values. The calculated modulus of elasticity for the smooth specimens was 195 GPa, which is accurate in regard to the expected material property. However, the calculated modulus of elasticity for the notched specimens was 340 GPa, which is a fictitious value due to the specimen design and axial-strain-measurement method. This error in the measured modulus of elasticity of the notched specimen is expected to be due to the limited region of elongation due to the localized elongation at the notched tip and the minimal elongation along the 9.53 mm gauge diameter. Thus, the test values for the smooth specimens are reported with actual axial-strain values, while the test values for notched specimens are reported with fictitious axial-strain values.

As shown in Figure 6a, the material produces isotropic tensile responses with respect to the specimen build direction regardless of the specimen geometry. It is noted that all of the notched specimens have much higher ultimate-stress levels in comparison to the smooth specimens. The increase in the ultimate-stress values of the notched specimens is likely associated with the higher level of triaxial stress within the notched specimens, causing additional material constraints, as reported in [15]. It is also seen that the smooth specimens exhibit a much higher level of elongation prior to failure, while the notched specimens exhibit failure with little-to-no elongation. 

A modified strain scale of the stress vs. axial-strain curve is shown in Figure 6b to provide some additional insight on the variation with notch sizes. It is noted that all of the notches have similar ultimate-stress levels. While the ultimate-stress value is insensitive to the notch root radius, there is an impact on elongation prior to failure. As the notch root radius increases, the amount of elongation prior failure also increases. This trend in elongation is also expected to be directly related to the amount of triaxial stress located near the notch tip. Smaller root radii are expected to exhibit higher levels of triaxial stress, reducing the total elongation within the test specimens.

**Figure 7:**
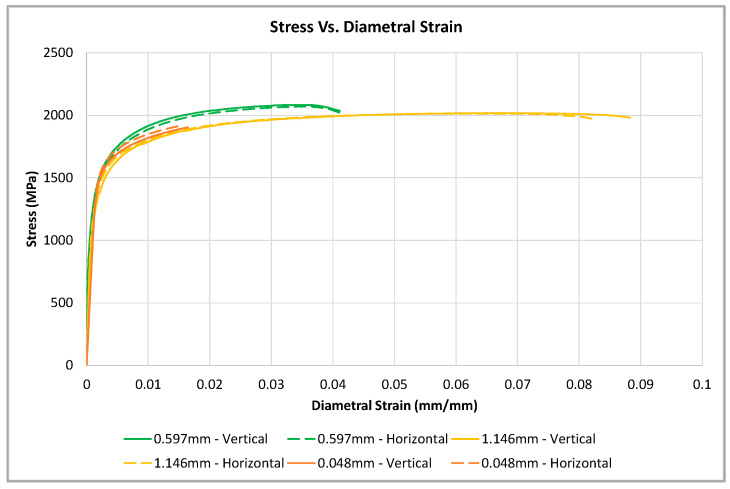
Stress vs. strain curves for the smooth and notched specimens produced in the vertical and horizontal directions. Axial-strain values were measured for the smooth specimens, while diametral strain values were measured for the notched specimens.

A typical stress vs. strain curve for each specimen type, tested with diametral strain measurements, is plotted on the stress vs. strain curve, as shown in Figure 7. Similar to the axial measured test results, the material produces isotropic tensile responses with respect to the specimen build orientation regardless of specimen geometry. Additionally, as the notch root radius increases, the notch root diameter undergoes additional reduction in size prior to failure. This increase in diametral strain is similar to the increase in the axial strain noted in Figure 6, and is also expected to be directly related to the amount of triaxial stress located near the notch tip.

### 3.2. Finite Element Analysis

**Figure 8:**
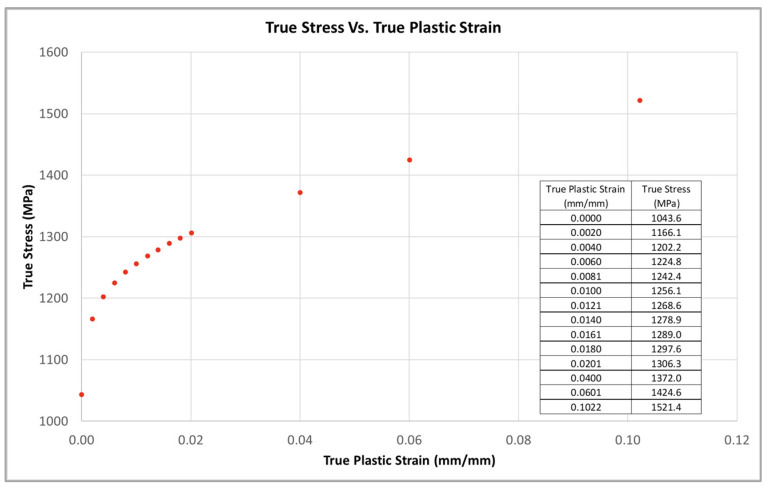
Multilinear isotropic hardening dataset (true stress vs. true plastic strain) derived from smooth specimen tensile test data.

FEA was completed on each specimen geometry configuration using the Ansys Mechanical. Isotropic elasticity and multilinear isotropic hardening properties were used to evaluate the specimens. All input mechanical property data were derived from the smooth specimen tensile test data. A Young’s modulus value of 195 GPa and Poisson’s ratio of 0.3 were used for the isotropic elasticity data. Additionally, a tabulated true stress and true plastic strain dataset was extracted from the actual smooth specimen tensile test data and added to the FEA multilinear isotropic hardening dataset. The true stress and true plastic strain dataset that is used in the FEA is shown in Figure 8.

**Figure 9:**
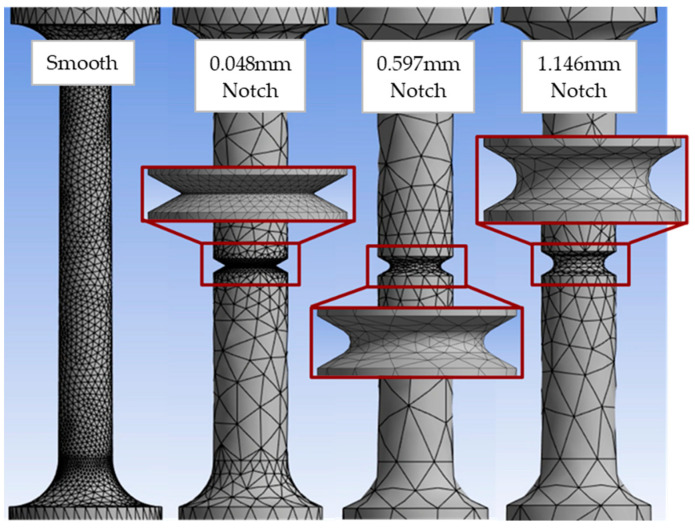
Finite element analysis (FEA) mesh of smooth and notched specimens.

Specimens were meshed using the Ansys default settings. Mesh refinement was added to the gauge section of the smooth specimen and the notch features of the notched specimens, as shown in Figure 9.

**Figure 10:**
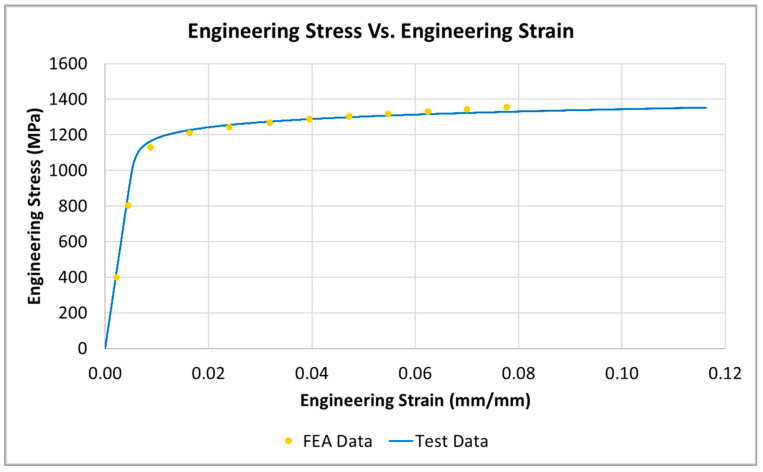
Engineering stress vs. engineering strain of the smooth specimen (FEA prediction and actual test data).

Initially, the smooth specimen was evaluated to validate the FEA material property dataset. Figure 10 compares the engineering stress vs. engineering strain data predicted by the FEA compared to the actual measured engineering stress vs. engineering strain test data. Results show that the FEA model has an acceptable correlation with the actual test data.

**Figure 11:**
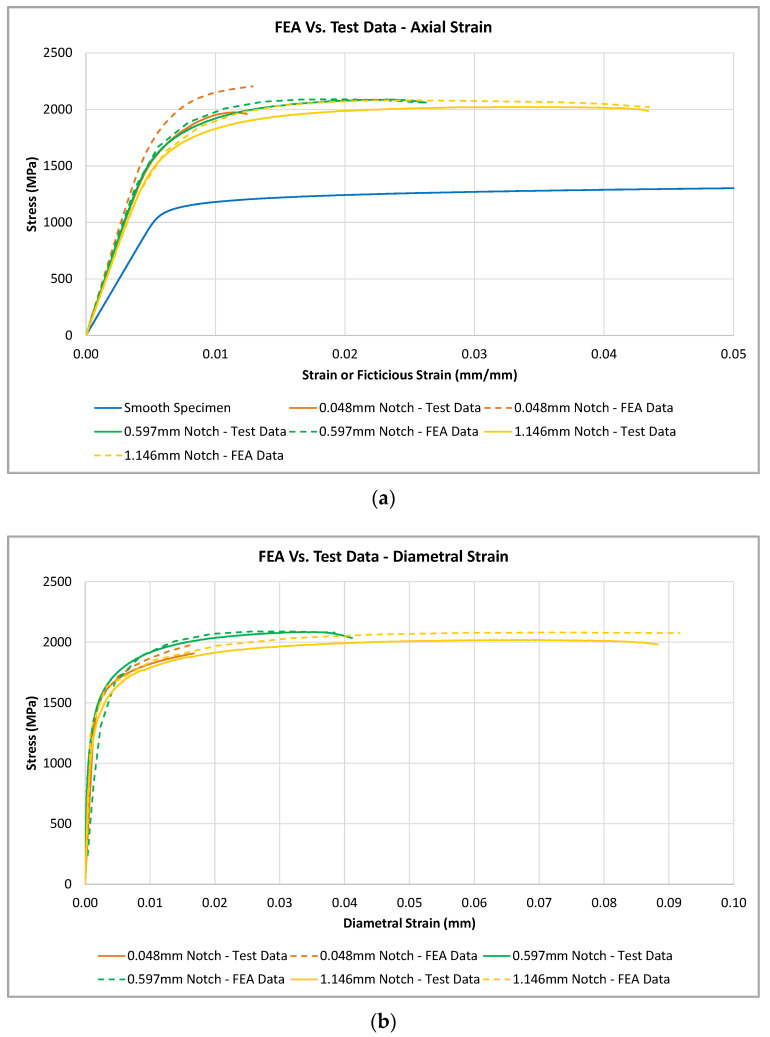
Comparison between FEA-generated stress vs. strain curves with actual test data of each notched geometry: (**a**) Axial strain FEA results; (**b**) Absolute diametral strain FEA results.

Following an initial validation of the FEA model with the smooth specimen geometry, the tensile performance was evaluated for each notch specimen. Engineering stress values were calculated based on the load applied to the specimen and the original diameter of the notched region. The axial strain was measured by evaluating the displacement of two nodes along the gauge length, while the axial strain was measured by evaluating the radial displacement at the notch root. The results of the notched specimen’s FEA are shown in Figure 11a for the axial-strain measurements and Figure 11b for the diametral-strain measurements. Additionally, the resulting FEA-generated stress vs. strain curves are compared to actual test data for the corresponding notch geometry in Figure 11a,b.

Results in Figure 11 show that the FEA data closely correlate to the actual test data. However, the FEA results tend to predict a slightly higher stress value for the 0.048 mm notch. This is likely due to difficulties in accurately machining the 0.048 mm notch. It is assumed that small deviations in the 0.048 mm machined notch compared to the nominal model used for the FEA analysis will have a large impact in the resulting test data and could have caused the variation shown. Figure 11a also shows that the fictitious modulus of elasticity values measured during testing and predicted during FEA analysis are equivalent. Additionally, Figure 11b shows the diametral modulus of elasticity values are equivalent when comparing the FEA predicted values and the actual test data.

### 3.3. Notch Sensitivity

A notch-sensitivity ratio (*NSR*) was used in this study to quantify the impact notches on the L-PBF Inconel 718 material. The *NSR* equation shown below in Equation (1) has been used in the past to investigate the notch sensitivity of high-temperature alloys and the embrittlement of high-strength materials [23,24]. The tensile strength of the notched tensile specimens (*σ*_(*n*)_) and the tensile strength of the smooth tensile specimens (*σ*_(*s*)_) were used to calculate the *NSR*. An *NSR* value less than one is found in brittle materials, a value equal to one indicates the notch insensitivity, and values greater than one indicate notch strengthening [25]. *NSR* values greater than one are typically found in ductile crystalline materials [13].

(1) $$NSR=\frac{\sigma_{\left(n\right)}}{\sigma_{\left(s\right)}}$$

In addition, the stress-concentration factor (*K_t_*) was calculated for each notch geometry, as shown in Equation (2) [23]. In order to calculate the *K_t_* value, the specimen’s notch depth (*a*) and notch root radius (*ρ*) were used.

(2) $$K_{t}=1+2\sqrt{\frac{a}{\rho }}$$

From Figure 6, it was shown that the tensile strength of the smooth test specimens was 1368 MPa in the vertical direction and 1372 MPa in horizonal direction. The calculated *K_t_* factor and *NSR* are shown in Table 3. The *NSR* was calculated using the noted tensile strengths for the smooth specimens and the notched tensile strengths listed in Table 3. It is noted that the *K_t_* values of the notches range from 3.35 up to 12.50. The *NSR* values were consistent regardless of specimen orientation or notch root radius and ranged from 1.44 up to 1.52, with an average value of 1.48. 

### 3.4. Stress vs. Strain Approximations

**Figure 12:**
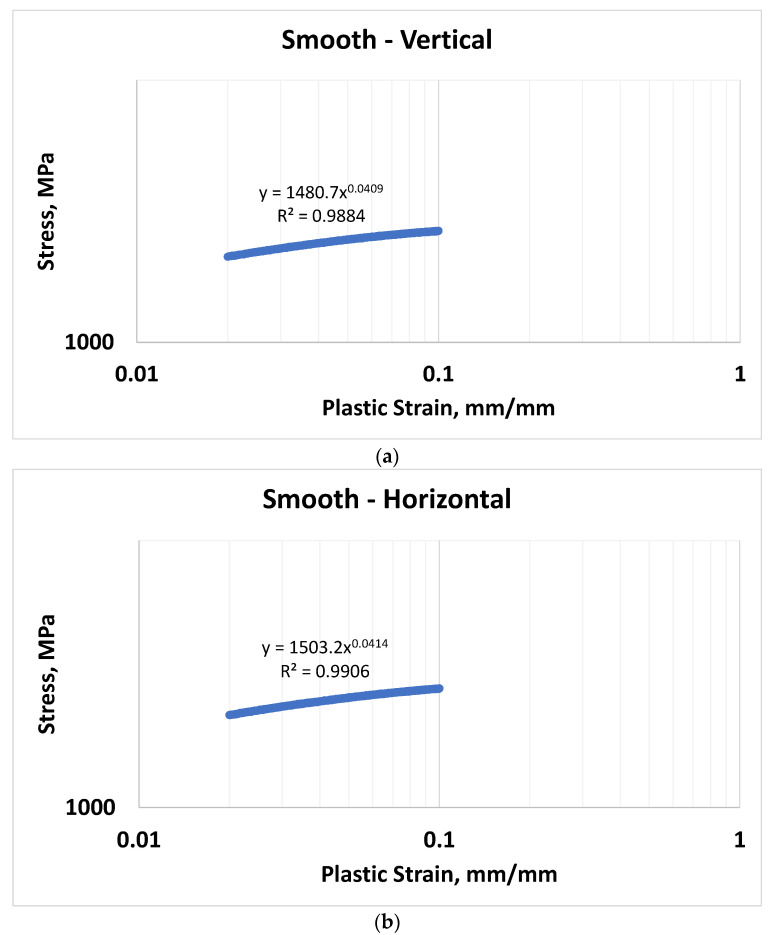
Ramberg–Osgood constants for vertical- and horizontal-oriented material: (**a**) Vertical orientation; (**b**) Horizontal orientation.

The Ramberg–Osgood equations were developed for both the vertical and horizontal orientations of the L-PBF Inconel 718 material, where ε is the material total strain, *σ* is the stress value associated with the given strain value, and *E* is the modulus of elasticity within the material. Figure 12 shows the stress vs. plastic-strain plots used to determine the material constants. Equation (3) represents the Ramberg–Osgood relationship for vertically tested material, while Equation (4) represents the Ramberg–Osgood relationship for horizontally tested material.

Ramberg–Osgood relationship (vertical orientation):

(3) $$\varepsilon =\frac{\sigma }{E}+{\frac{\sigma }{1480.7}}^{\frac{1}{0.0409}}$$

Ramberg–Osgood relationship (horizontal orientation):

(4) $$\varepsilon =\frac{\sigma }{E}+{\frac{\sigma }{1503.2}}^{\frac{1}{0.0414}}$$

**Figure 13:**
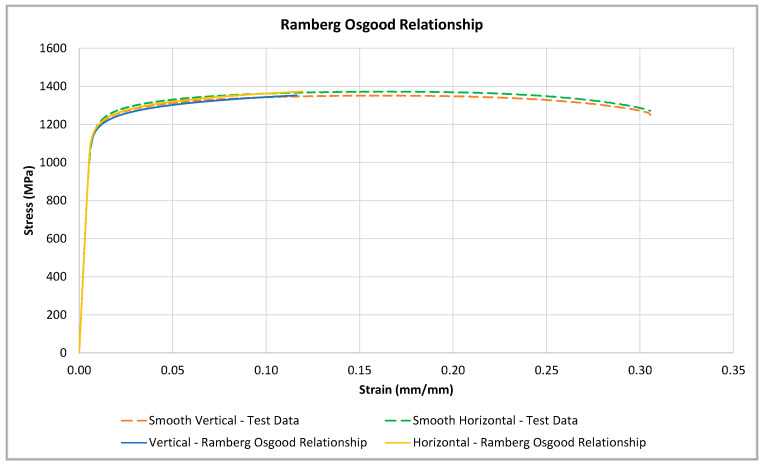
Ramberg–Osgood approximation for vertical- and horizontal-oriented material.

The Ramberg–Osgood relationships defined by Equations (3) and (4) are plotted and compared to actual test data within Figure 13. Figure 13 shows that the Ramberg–Osgood relationships have a good fit with respect to the actual test data for both the vertical and horizontal orientations.

Neuber and Glinka approximations were used to estimate the stress within the notch and compared to the actual test results. The Neuber relationship was calculated using Equation (5), shown below. The Glinka relationship was derived by setting the strain energy density with the absence of yielding (*W_e_*) equal to the strain energy density with the presence of yielding (*W_p_*), as shown in Equation (6). Equation (7) is derived from Equation (6) by substituting the area underneath the stress–strain curves for the linear elastic scenario (*W_e_*) and the area under the Ramberg–Osgood approximation for *W_p_*, where *S* is equal to the nominal stress, *n* is equal to the strain hardening exponent, and *H* is equal to the strength coefficient.

Neuber relationship:

(5) $$\sigma \cdot \varepsilon =\frac{{\left(K_{t}\cdot S\right)}^{2}}{E}$$

Glinka relationship:

(6) $$W_{e}=W_{p}$$

(7) $$\frac{{\left(K_{t}\cdot S\right)}^{2}}{2\cdot E}=\frac{\sigma^{2}}{2\cdot E}+\frac{\sigma }{1+n}\cdot {\left(\frac{\sigma }{H}\right)}^{\frac{1}{n}}$$

**Figure 14:**
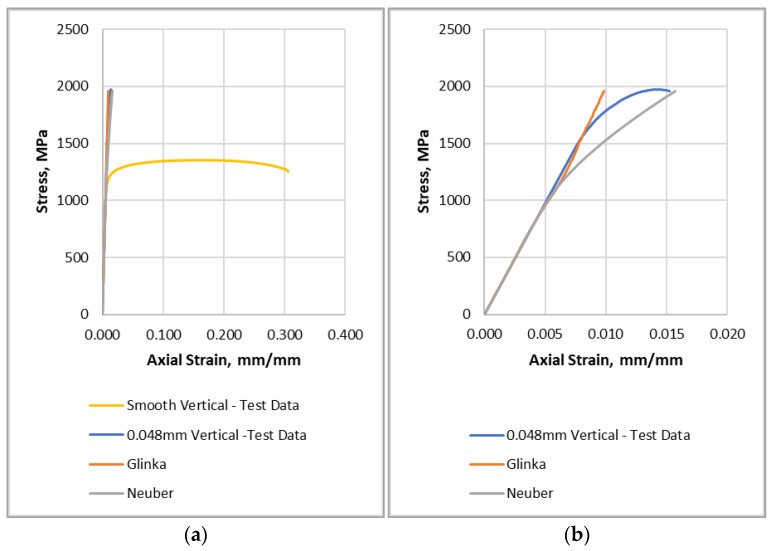
Neuber and Glinka notch stress approximations: (**a**) Entire axial-strain scale; (**b**) Reduced axial-strain scale to evaluate differences in the approximation methods.

Equations (5) and (7) were plotted and compared to the smooth vertical specimen test data and the vertical 0.048 mm notch data in Figure 14. Figure 14b shows the stress–strain curves near the linear elastic region to evaluate the differences between the approximation methods. From Figure 14b, it is shown that the Neuber method overestimates strain in comparison to the Glinka method for a given stress value.

### 3.5. Fractography

**Figure 15:**
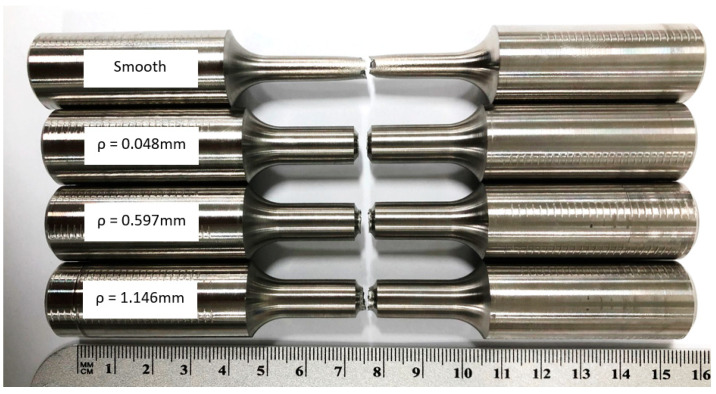
Fractured L-PBF Inconel 718 smooth and notched tensile specimens.

Evaluation of the fractured surfaces can provide additional insight to understanding the amount of notch sensitivity within a material. The driving failure modes of the tested material may be revealed and compared to each other. The fractured surfaces of the tested tensile specimens in this study were evaluated using optical microscopy. A Keyence digital microscope was used to obtain the 3D images presented in this section. The general fracture evaluation of the smooth specimens and each notch specimen is shown in Figure 15. It can be seen that the smooth specimen exhibits some degree of visual elongation and the reduction of cross-sectional area, while the notch specimens appear to exhibit minimum elongation. Some minor elongation in the notched region can be observed on the 1.146 mm root radius; however, the 0.048 mm root radius visually exhibits no elongation.

Previous studies have shown that the elongation is determined by dislocations slipping over the entire gauge length of the entire smooth specimen, while the notched specimens only have a minimum length for the dislocation slippage to stake up and provide a significant contribution to the elongation measurements. As a result, the smooth specimens have significantly more elongation than the notched specimens, and significant necking is able to occur prior to fracture within the smooth specimen.

**Figure 16:**
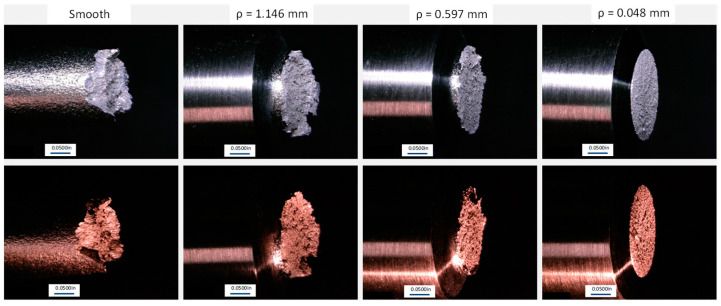
General optical images of the fracture surfaces.

Figure 16 provides general optical images of the fracture surface. It can be seen that both the smooth and the 1.146 mm notch specimens’ fracture surface display a shear lip all the way around the specimens, indicating a macroductile failure mode. The fracture surfaces of the 0.597 mm and 0.048 mm specimens exhibit no shear lip, indicating a more macrobrittle fracture mode when compared to the smooth and 1.146 mm notched specimens. It can also be seen that the smooth specimen demonstrates evidence of elongation and the reduction of the area due to the indications on the outside diameter of the specimen near the fracture surface. 

**Figure 17:**
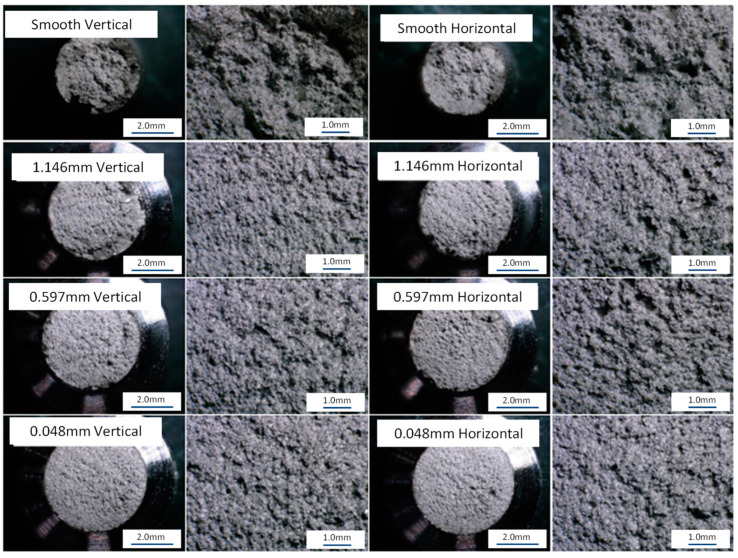
Fractured L-PBF Inconel 718 smooth and notched tensile specimens. Fracture surfaces at 30× and 100× magnification.

**Figure 18:**
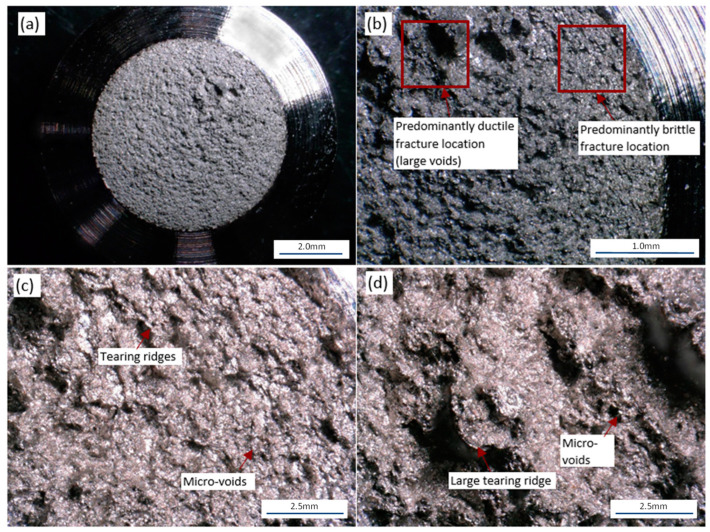
Fracture surface of a 0.048 mm notched vertical specimen: (**a**) Notched fracture surface at 30× magnification; (**b**) Notched fracture surface at 100× magnification; (**c**) Edge of a notch fracture surface at 300× magnification; (**d**) Center of a notch fracture surface at 300× magnification.

Figure 17 provides general fractured surface images of each notch geometry and build direction. No significant difference in the fracture surfaces were noted due to the build direction. It is noted that the smooth specimens exhibited a large degree of ductile failure modes. The fracture surface of the smooth specimens shows a significant level of dimpled surfaces and microvoid coalesces. This high level of ductility has a strong relationship with the elongation noted from the stress–strain curves. The failure mode observed for the notched specimens is significantly different than the failure modes observed in the smooth specimens. In comparison to the smooth specimens, the notched specimens have a less ductile failure-mode fracture surface. In addition, the notched specimens have a mixed mode of ductile and brittle failure locations within the fracture surface. As shown in Figure 17, the notched specimens have a visually smoother fracture surface around the edges of the fracture surface and a visually rougher surface near the centers of the specimens. The mixed mode of the fracture surface noted in Figure 17 was investigated further at higher magnifications in Figure 18. Figure 18c shows the outer edge of the fracture surfaces, where small dimples and tearing ridges can be noted. In comparison, Figure 18d shows significantly larger dimples, tearing ridges, microvoid coalesces, and cavities, indicating a higher degree of ductility. 

**Figure 19:**
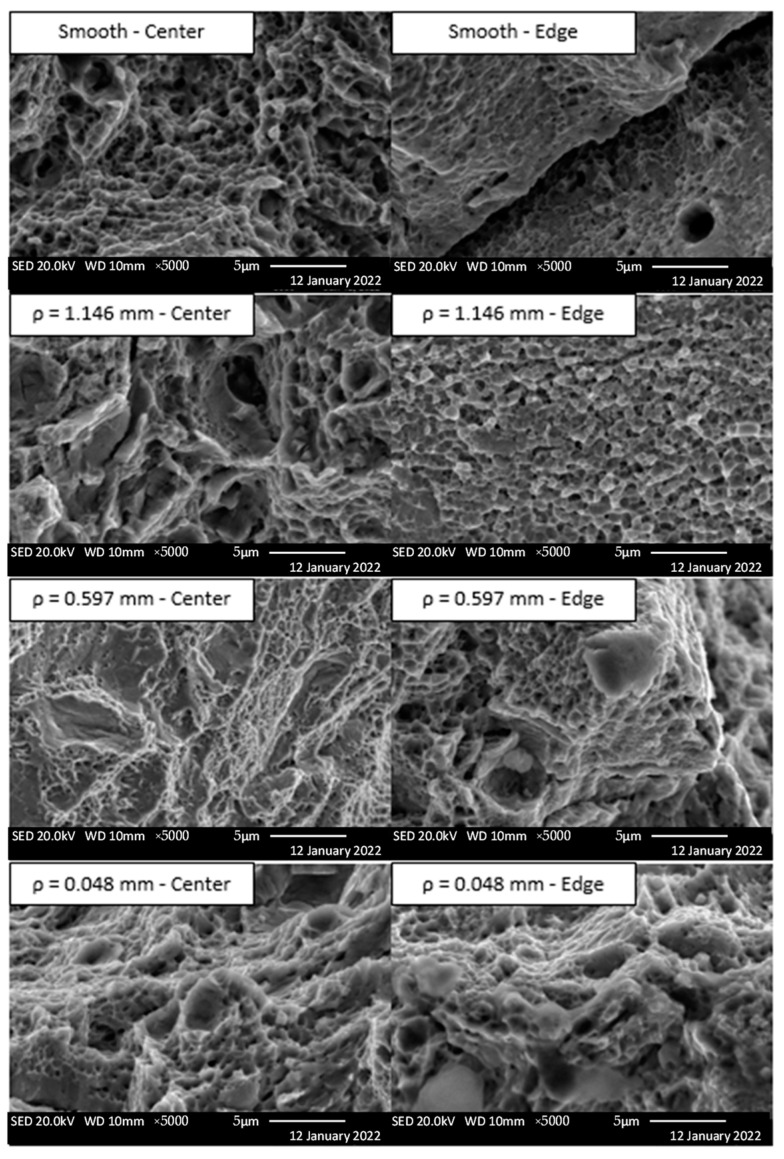
SEM secondary electron images.

Furthermore, scanning electron microscope (SEM) evaluation was completed using a JEOL JSM-IT100 SEM of each specimen configuration. Figure 19 shows the SEM images at 5000× near the center and near the edge of the fracture surfaces. Very fine microvoid coalesces were seen near the edge of the smooth and 1.146 mm notched specimens along the shear lip. The centers of the smooth and 1.146 mm notched specimens exhibit larger microvoid coalesces, cavities, and tearing ridges, all of which are associated with a ductile failure mode. Both the 0.597 mm and 0.048 mm notched specimens exhibited fine microvoid coalesces throughout the fracture surface, indicating a more uniform fracture. In summary, the 1.146 mm notched specimens appear to have a higher degree of ductility within the fracture surface when compared to both the 0.597 mm and 0.048 mm notched specimens. The smaller root radii specimens in general are more brittle; however, they still are classified as ductile failures.

## 4. Conclusions

This study investigated the impact of V-notches on Inconel 718 produced by L-PBF. The impact of the notches was investigated in both the vertical and horizontal build directions and included three different notch root radii. The L-PBF Inconel 718 material has demonstrated that it exhibits notch strengthening under room-temperature monotonic-loading conditions. Based on these results, the following conclusions can be drawn:

1. The tested L-PBF Inconel 718 material has isotropic room-temperature tensile-material performance. No significant difference was noted in the tensile data when comparing the vertical and horizontal build directions;
2. The *NSR* for the notched specimens was independent of the notch root radii and *K_t_*. It was determined that the average *NSR* of the notched specimens was 1.48. Since the *NSR* was found to be greater than one, it was determined that the L-PBF Inconel 718 material exhibited notch strengthening;
3. The smooth specimens exhibited much higher levels of elongation due to the increase in the area for dislocations to occur and stack up, while the notched specimens only had a short length for dislocations to occur, and thus had a minimum amount of elongation prior to failure;
4. The notched specimens exhibited a much higher ultimate tensile strength when compared to the smooth specimens. The cause of the increased ultimate tensile strength is expected to be similar to the reasonings reported within [15]. The increase in the ultimate tensile strength of the notched specimens is suspected to be associated with the higher level of triaxial stress near the notch tip. The triaxial stress near the notch tip produces a localized area of plastic deformation that is constrained by the bulk material. Due to this added constraint, higher stresses are required to produce failure.

## Figures and Tables

**Table 1:** Chemical composition of the laser power bed fusion (L-PBF) Inconel 718 powder.

Element	Composition (wt. %)
Aluminum	0.53
Boron	0.002
Calcium	<0.005
Carbon	0.034
Chromium	18.4
Cobalt	0.023
Copper	0.032
Iron	18.05
Molybdenum	2.84
Nickel	54.0
Niobium	4.94
Nitrogen	0.0098
Oxygen	0.020
Silicon	0.040
Sulfur	0.002
Titanium	0.98

**Table 2:** Tensile test matrix; all tests were conducted at room temperature.

Specimen Type	Specimen Build Orientation	Test Quantity (Axial Strain)	Test Quantity (Diametral Strain)
Smooth	Vertical Horizontal	3 3	2 2
*ρ* = 0.048 mm	Vertical Horizontal	3 3	2 2
*ρ* = 0.597 mm	Vertical Horizontal	3 3	2 2
*ρ* = 1.146 mm	Vertical Horizontal	3 3	2 2

**Table 3:** Stress-concentration factor and notch-strength ratio of L-PBF Inconel 718.

Orientation	Vertical	Horizontal	Vertical	Horizontal	Vertical	Horizontal
Notch Root Radius, *ρ* (mm)	0.048	0.048	0.597	0.597	1.146	1.146
*K_t_*	12.50	12.50	4.26	4.26	3.35	3.35
*R_m_*_(*N*),_ (MPa)	1970	1985	2082	2082	2021	2007
*NSR*	1.44	1.45	1.52	1.52	1.48	1.46

## Data Availability

The data presented in this study are available on request from the corresponding author.
